# *In Vitro* Effects of St. John’s Wort Extract Against Inflammatory and Oxidative Stress and in the Phagocytic and Migratory Activity of Mouse SIM-A9 Microglia

**DOI:** 10.3389/fphar.2020.603575

**Published:** 2020-12-03

**Authors:** Gabriel A. Bonaterra, Olga Mierau, Johanna Hofmann, Hans Schwarzbach, Heba Aziz-Kalbhenn, Christiane Kolb, Ralf Kinscherf

**Affiliations:** ^1^ Department of Medical Cell Biology, Anatomy and Cell Biology, University of Marburg, Marburg, Germany; ^2^ Bayer Consumer Health Division, Phytomedicines Supply and Development Center, Steigerwald Arzneimittelwerk GmbH, Darmstadt, Germany

**Keywords:** anti-depressive, cortisol, *Hypericum perforatum* L, NMDA/glutamate toxicity, STW3-VI, anti-inflammatory, microglia, St. John’s wort

## Abstract

**Introduction:** Herbal medicinal plants as *Hypericum perforatum* L., known as St. John’s wort (SJW) have been in use for a long time. SJW that is specifically used for the treatment of depressive disorders. Inflammatory cytokines derived from microglia play an important role in the regulation of the synthesis and reuptake of glutamate and influence synaptic function, morphology and neuronal plasticity. The present study was performed to investigate, whether STW3-VI, a special SJW extract has protective effects on mouse SIM-A9 microglia against cytotoxic and proinflammatory effects of ROS, glutamate, NMDA or cortisol. Additionally, we investigated the effects of SJW on migratory and phagocytic properties of microglia.

**Results:** Pre-treatment (48 h) of microglia with STW3-VI (5 or 10 μg/ml)—in contrast to desipramine—inhibited the H_2_O_2_-induced TNF-α release by 20–40%. Pre-treatment (48 h) of microglia with STW3-VI (5 or 10 μg/ml) delayed the 3 or 4 mM H_2_O_2_-induced intracellular ROS level by 26.9 and 44.4%, respectively. Furthermore, pre-treatment (48 h) of microglia with STW3-VI (5 μg/ml) - in contrast to desipramine - lowered the glutamate-induced cytotoxicity by 13.2%. Besides, pre-treatment (48 h) of microglia with STW3-VI (5 or 10 μg/ml) or desipramine (5 µM) inhibited the NMDA-induced decrease of the viability by 16.5–28.8% or 12%, respectively. Finally, pre-treatment (48 h) of microglia with STW3-VI (5 or 10 μg/ml)—in contrast to desipramine - reduced the cortisol-induced cytotoxicity by 15.5 and 12.9%. Treatment of microglia with STW3-VI (10 or 100 μg/ml) increased the migratory and the phagocytic capacities by 100 and 40%.

**Conclusion:** Our data provide evidence that STW3-VI—in contrast to desipramine - protects microglia from oxidative stress, NMDA- or glutamate-induced cytotoxicity, and has anti-inflammatory properties that are accompanied by improvement of their migratory and phagocytic capacity. These protective (particularly the anti-inflammatory) properties may be beneficial in the treatment of depressive disorders.

## Introduction

Stress is a normal part of everyday life. When stress becomes chronic it can trigger and develop psychological issues such as depression (Lazaratou et al., 2010). When the stress response system is activated, this affects other systems, increasing heart rate, blood pressure, blood sugar levels, and decreasing immune responses. Chronic stress causes deregulation of body systems and therefore, progressively damage them. Three theories relating to the development of mood disorders involve activation of inflammatory pathways, alterations of glutamate metabolism, as well as the alteration of the neuronal plasticity Haroon et al. (2016) and Haroon et al. (2017). There are common mechanisms of cell injury, as glutamate-dependent toxicity, and inflammation, both playing an integral role in a variety of neurobiological disorders such as Parkinson’s disease, Alzheimer’s disease, and depression (Haroon et al., 2016; Haroon et al., 2017; Guo et al., 2020; Saitgareeva et al., 2020). Stressful life events may alter the function of the hypothalamic-pituitary-adrenal (HPA) axis and consequently the release of steroid hormones, glucocorticoids, and inflammatory cytokines (Dinan, 2009). These alterations have a direct effect on neurons and glial cells involved in neurogenesis, neuronal plasticity, and the development of neurological disorders such as depression (Duman et al., 2016; Singhal and Baune, 2017). In this context, microglia exhibit trophic functions and actively respond to the changes in neural activity (Gras et al., 2012) and engage in neuronal pruning to maintain synaptic and functional specialization during neurogenesis as well as neuronal plasticity (Schafer and Stevens, 2015; Matcovitch-Natan et al., 2016). Moreover, microglia have an important role in the regulation of the synthesis and reuptake of glutamate and thereby influence synaptic function and morphology (Popoli et al., 2012). Additionally, inflammatory cytokines derived from microglia, influence synaptic plasticity, and the synapse formation at physiological conditions (Khairova et al., 2009; Chen et al., 2020). The brains of patients with mood disorders or depression have shown signs of activated microglia (Haroon et al., 2017; Chen et al., 2020). Characteristics and possibly also cause of neurodegenerative diseases are chronic over-activation of microglia and increase of oxidative stress (Haroon et al., 2017; Chen et al., 2020). Therefore, it is beginning to be accepted that reactive oxygen species (ROS) produced by either microglia or the surrounding cellular environment not only impact neurons, but also modulate activity of microglia (Rojo et al., 2014; Fischer and Maier, 2015). A large body of research has documented the role of microglia in neurodegenerative disorders. Primary or transformed immortalized microglia cultures are useful tools to investigate microglial behavior *in vitro*. In this context, spontaneously differentiated mouse SIM-A9 microglia cell line is expected to behave more comparably to primary microglia than virally transformed cells (Nagamoto-Combs et al., 2014) and has been recently used as a model of activated microglia in the context of neuropathic pain (Dave et al., 2020). However, function of microglia seems to be a double-edged sword, because on the one hand they may inhibit neuroinflammation, with a beneficial effect on eliminating cell debris, tissue healing, and repair, but on the other hand the chronic activation of these cells may cause harmful effects on neurons (Perry et al., 2010; Saitgareeva et al., 2020). This implicates that microglia may be a potential target for the treatment of these diseases. In this context St John’s wort (SJW) may interfere with a wide variety of signaling processes in the brain, which are involved in the pathogenesis of stress-related neurobiological disorders (Mahar et al., 2014; Cattaneo et al., 2015). By using *in vitro* and *in vivo* models of Alzheimer’s disease, Hofrichter et al. showed that SJW extracts decrease the number and size of amyloid plaques, rescue neurons, alleviate memory impairments, and activate microglia (Hofrichter et al., 2013). STW3-VI is a clearly defined SJW dry extract, which fulfills the requirements of the European Pharmacopeia (European Pharmacopoeia, 2008) and is commercially available as marketed medicinal product, e.g., in Germany (Laif^®^900, Steigerwald Arzneimittelwerk GmbH, Darmstadt, Germany). Most recent data have shown that the STW3-VI stimulates the neurite formation in differentiated hippocampal neurons and has protective effect against glutamate or N-methyl-D-aspartate (NMDA) induced-cytotoxicity (Bonaterra et al., 2018). Moreover, regarding antioxidant defenses, STW3-VI increased the intracellular reduced glutathione contents compared with the effect of glutamate or NMDA; additionally, STW3-VI has shown anti-inflammatory properties against lipopolysaccharide (LPS)-induced macrophage activation (Bonaterra et al., 2018). These protective, neurotrophic, and anti-inflammatory properties indicate the suitability of SJW/STW3-VI in the treatment of disorders like e.g., depression (Mahar et al., 2014; Cattaneo et al., 2015; Bonaterra et al., 2018). Thus, the aim of our *in vitro* investigations was to determine the protective effects of STW3-VI on the mouse SIM-A9 microglia against the cytotoxic and proinflammatory effects of ROS, i.e., H_2_O_2_ as well as the cytotoxicity induced by glutamate, NMDA or cortisol. Additionally, we wished to investigate the effect of STW3-VI on the migratory and phagocytic properties of microglia. Because desipramine has already been used as a control by us and several other groups (e.g., Lopez et al., 1998; Jakobs et al., 2013; Bonaterra et al., 2018), this drug was chosen as a reference control in the present study, too.

## Material and Methods

### Tested Substances

Steigerwald Arzneimittelwerk GmbH (Darmstadt, Germany) provided the SJW dry extract STW3-VI of *Hypericum perforatum* L., according to Kew Medicinal plant names service. The characterization and methods of extraction have been previously described (Breyer et al., 2007; Grundmann et al., 2010; Jungke et al., 2011).

The analysis of STW3-VI (dry extract, lot 14-0155) shows that the drug contains 0.23% of total hypericin, 1.83% of hyperforin, 9.19% of flavonoids (calculated as rutin), residual 0.048% ethanol and 3.8% water, according to the principles as described in chapter “reference standards” of the European Pharmacopoeia (2008). The corresponding TLC fingerprint and HPLC analyses of the main components of STW3-VI, i.e., flavonoids, hypericin and hyperforin (which are used for internal standardization), are shown in the Supplementary Figures S1‒S3. The lot 14-0155 (used in the present study), originates from the same preparation as previously published by Bonaterra et al. (2018). The drug extract ratio (DER) was 3–6:1 and the extractant was 80 Vol. % Ethanol. Moreover, the antidepressant desipramine hydrochloride, [desipramine, (CAS no. 58-28-6. Merck/Sigma-Aldrich Chemie GmbH, Munich, Germany) was used as a control as previously described by us and others (e.g., Lopez et al., 1998; Jakobs et al., 2013; Bonaterra et al., 2018).

### Cell Culture

The murine brain spontaneous immortalized mouse microglia (SIM-A9 [ATCC^®^ CRL-3265^™^]; Nagamoto-Combs, et al., 2014), were cultured at 37°C in humified CO2 (5%) in Dulbecco’s Modified Eagle’s Medium (DMEM): F-12 Medium, a 1:1 mix of DMEM and Ham’s F-12 supplemented with heat-inactivated 10% fetal bovine serum (FBS), 5% horse serum and penicillin/streptomycin [(100 U/ml, 0.1 mg/ml) (Capricorn Scientific GmbH, Ebsdorfergrund, Germany)].

### Measurements of the Viability and Survival of Mouse SIM-A9 Microglia

Mouse SIM-A9 microglia (3 × 10^4^ cells × well) were seeded in 96-well microtiter plates (BD Falcon^™^ Becton Dickinson GmbH, Heidelberg, Germany) and were allowed to attach to the plate surface by growing in supplemented DMEM: F-12 Medium overnight, afterwards the medium was changed and the cells were pre-treated during 24 or 48 h with different concentrations of STW3-VI (5, 10, 20, 40, 80, 120, and 160 μg/ml) or desipramine (5, 10, 20, 40, 60, and 80 µM) as reference drug (Merck/Sigma-Aldrich Chemie GmbH). As a control (= 100% viability), we used cells cultured with medium alone (untreated control). Cell viability was measured by using PrestoBlue^®^ reagent (Fisher Scientific GmbH, Schwerte, Germany) directly added into the culture medium at a final concentration of 10% and measured according to the manufacturer’s specifications. PrestoBlue^®^ is a cell viability indicator based on mitochondrial enzyme activity (Xu et al., 2015). Results were expressed in % of viability = (OD_570 nm/600 nm_ of samples x 100)/OD _OD570 nm/600 nm_ of untreated control.

### Measurement of the TNF-α Release

The release of TNF-α was quantified using enzyme-linked immunosorbent assay (ELISA). In detail, 3 × 10^4^ SIM-A9 cells were seeded in 100 ml medium/well using 96-well plates (Falcon™, BD Bioscience); thereafter, the medium was changed and the SIM-A9 were pre-treated 48 h with 5 or 10 μg/ml STW3-VI or 5 or 10 µM desipramine alone and afterwards 24 h co-incubation with or without 3 mM H_2_O_2_ or 4 mM hydrogen peroxide [H_2_O_2_ (CAS no. 7722-84-1, Merck/Sigma-Aldrich)]. TNF-α was determined in the culture medium using the assay DuoSet ELISA Development kit (R&D Systems Europe, Ltd., Abingdon, UK) following the kit’s instructions. The reaction was performed in NUNC MaxiSorp™ (Thermo Fisher Scientific, St. Leon-Rot, Germany) 96 wells microplates using 50 μl peroxidase substrate Sigma Fast™ o-phenylenediamine dihydrochloride [OPD, (CAS no. 615-28-1 (Merck/Sigma-Aldrich)] 30 min; at room temperature RT. The reaction was stopped with 25 μl 3 N hydrochloric acid [HCl, (CAS no. 7647-01-0, Merck/Sigma-Aldrich] per well. The absorbance was measured at 490 nm and 655 nm as a reference. Afterwards, the cells were fixed with 4% paraformaldehyde [PFA, (CAS no. 30525-89-4, Merck/Sigma-Aldrich)] in PBS and stained with crystal violet (CAS no. 548-62-9 Merck/Sigma-Aldrich) solution (0.04% crystal violet in 4% ethanol [v/v]) and washed with PBS; then, the cells were lysed in a 1% sodium dodecyl sulfate [SDS, (CAS no. 151-21-3 Merck/Sigma-Aldrich)] solution and thereafter the absorbance was measured at 595 nm and 655 nm as a reference to spectrophotometrically determine the total cell number. The amount of TNF-α released into the medium was normalized against crystal violet absorbance.

### Determination of Reactive Oxygen Species

The production of intracellular ROS was determined by measuring the fluorescence intensity of the oxidant-sensitive 2′-7′-dichlorofluorescein diacetate [DCFDA, (CAS no. 4091-99-0 Merck/Sigma-Aldrich)]. In detail, 3 × 10^4^ SIM-A4 cells were seeded in 100 ml medium/well using 96-well plates (Falcon™, BD Bioscience); thereafter, the medium was changed and the SIM-A9 were pre-treated 24 or 48 h with 5 or 10 μg/ml STW3-VI or 5 or 10 μg/ml desipramine (Merck/Sigma-Aldrich) alone and afterwards 24 h co-incubation with or without 3 mM H_2_O_2_ or 4 mM H_2_O_2_. ROS was detected by using 10 mM of the fluorescent DCFDA incubated during 30 min and measured after 24 and 48 h. Total ROS was quantified, considering the fluorescence intensity (relative fluorescence units, RFU), measured at 495 nm excitation/529 nm emission and at the end the cells were stained with 5 μg/ml Hoechst 33342 (Thermo Fisher Scientific GmbH, Schwerte, Germany) and the RFU (RFU of cell nuclei) was measured at 350 nm excitation/461 nm emission using the Cytation™ 3 Cell Imaging Multi-Mode Reader (BioTek Instruments, Inc., Winooski, United States). Total intracellular ROS DCFDA-RFU was normalized with the bisbenzimide H33342 trihydrochloride [Hoechst 33342, (CAS no. 875756-97-1, Merck/Sigma-Aldrich)]-RFU of cell nuclei.

### Determination of the Migratory Capacity

To investigate the effect of STW3-VI on the migratory capacity of microglia, a transwell migration assay was conducted. In a 24-well plate, 750 µl medium was pipetted to each well and an insert provided with 8 μm pore polyethylene terephthalate (PET) membrane (Sarstedt AG & Co. Nümbrecht) was placed to each well. Mouse SIM-A9 microglia (5 × 10^4^ cells) were placed in the upper chambers of the insert and the lower chambers were filled with supplemented DMEM: F-12 medium and incubated 1 h. Then the cells were stimulated (24 h) with 5 or 10 μg/ml STW3-VI, 5 or 10 µM desipramine, or 20 ng/ml interleukin-4 (IL-4, R&D Systems Europe) as a positive control for migration. The cells that did not migrate to the lower side of the membrane were removed with a cotton bud and afterwards, the insert was washed carefully with PBS. Migrated cells were fixed with 500 µl 10% PFA/PBS (10 min; RT). An additional washing step with PBS was performed to remove the PFA before the nuclei of the cells were stained with 1 μg/ml DAPI (10 min; RT; in the dark). Then the insert membranes were photographed in the middle of the membrane and two images were taken with an Axiovert 135 microscope equipped with an AxioCam MRm Camera (Carl Zeiss, AG). These images were evaluated and quantified with the software Fiji (Schindelin, et al., 2012).

### Determination of the Phagocytic Activity

To determine whether STW3-VI stimulates mouse SIM-A9 microglia to increase their phagocytic activity, polystyrene fluorescent (FITC) FBS-opsonized microparticles 0.5–1.0 µm (Polysciences Inc. Eppelheim, Germany) were used. Therefore, 1.8 × 10^5^ cells/well were seeded in a 24-well plate and incubated (24 h) with 5, 10 or 100 μg/ml STW3-VI 5, 10 or 100 µM desipramine or 25 μg/ml LPS, (Merck/Sigma-Aldrich), afterwards the fluorescent microparticles were added and after 24 h the cells were washed five times with 2 ml PBS to eliminate the non-phagocyted microparticles Then the cells were fixed with 200 µl 10% PFA/PBS (15 min; RT) and washed two times with PBS. Thereafter, the nuclei were stained using 1 μg/ml DAPI (Thermo Fisher Scientific Inc.), 10 min (RT, in the dark). The fluorescence intensity of FITC or DAPI was measured with the Cytation™ 3 Cell Imaging Multi-Mode Reader (BioTek Instruments) at 485 nm excitation and 528 nm emission (FITC) and 360 nm excitation and 460 nm emission (DAPI). Total engulfed microparticles FITC-RFU were normalized with the DAPI-RFU.

### Statistical Methods

The software SigmaPlot®-12 (Systat Software GmbH, Erkrath, Germany) was used to perform statistical analyses by the unpaired Student's t‐test or Mann-Whitney U-test. Data are shown as mean + SEM. A p-value less than 0.05 ( p ≤ 0.05) was considered as statistically significant.

## Results

### Protective Effects of STW3-VI on H_2_O_2_-Induced TNF-α Release by SIM-A9 Microglia

**FIGURE 1:**
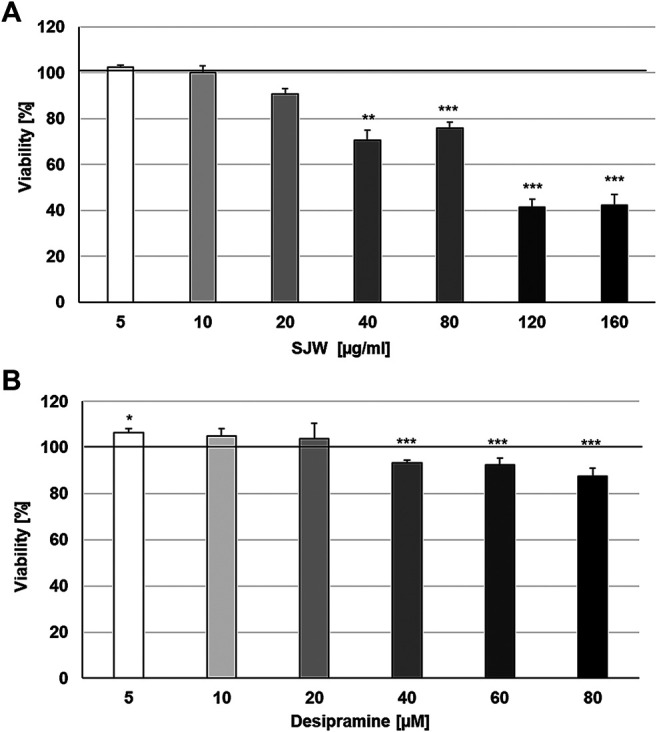
Effects of 48 h treatment with **(A)** STW3-VI [St. John’s wort (SJW)] extract or **(B)** desipramine on the viability of mouse SIM-A9 microglia. Viability was measured by PrestoBlue^®^ assay. Values [in % viability of untreated control (control = 100% viability)] are given as mean + SEM; significance (by T-TEST): **p ≤ 0.01 or ***p ≤ 0.001 vs. untreated control; n = 4.

First of all, we investigated the effect of 48 h treatment with STW3-VI or desipramine on the viability of SIM-A9 cells using PrestoBlue^®^ cell viability reagent, The treatment of mouse SIM-A9 microglia with 5, 10, and 20 μg/ml STW3-VI or 5, 10, and 20 µM desipramine did not affect their viability; however, at concentrations of 40, 80, 120, and 160 μg/ml STW3-VI inhibited the viability by 29.3‒57.7% and at concentrations of 40, 60, and 80 µM desipramine inhibited the viability by 6.6‒12.6% in comparison with the untreated control (Figures 1A, B).

**FIGURE 2:**
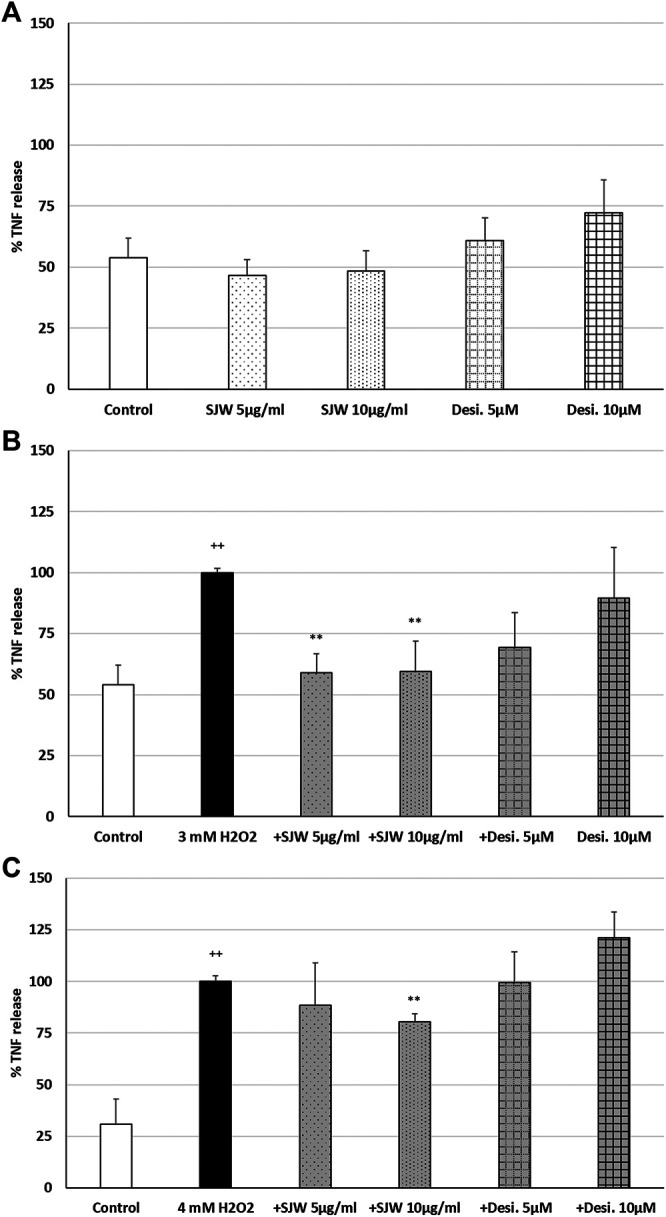
Effects of 48 h pre-treatment with STW3-VI [St. John’s wort (SJW)] or desipramine (desi.) and afterwards 24 h co-incubation with **(A)** SJW or desi. alone, **(B)** 3 mM H_2_O_2_ or **(C)** 4 mM H_2_O_2_ on the TNF-α release in mouse SIM-A9 microglia. Data are given as mean + SEM; significance (by T-TEST): **p < 0.01, ***p < 0.001 vs. 3 mM or 4 mM H_2_O_2_ treated cells; ^++^ *p* < 0.01 vs. untreated control; n = 5.

ROS may be a crucial hallmark in the development of depression, because they may trigger the development of pro-inflammatory microglial phenotypes (Rojo et al., 2014; Wenzel et al., 2020). According to this concept, we investigated the possible protective effects of STW3-VI against H_2_O_2_-induced inflammation in mouse SIM-A9 microglia, indicated by TNF-α release, which was measured after 48 h pre-treatment with STW3-VI or desipramine followed by co-incubation (24 h) with H_2_O_2_. The pre-treatment of mouse SIM-A9 microglia with STW3-VI (5 or 10 μg/ml) or desipramine (5 or 10 µM) alone did not significantly affect the TNF-α release compared with the untreated control (Figure 2A). Treatment of mouse SIM-A9 microglia with 3 mM or 4 mM H_2_O_2_ significantly (*p* ≤ 0.01) increased the TNF-α release by 46.1 and 69.1% compared with the untreated control (Figures 2B, C). However, pre-treatment of mouse SIM-A9 microglia with 5 or 10 μg/ml STW3-VI significantly (*p* ≤ 0.01) inhibited the H_2_O_2_ (3 mM)-induced pro-inflammatory TNF-α release by 40%, (Figure 2B), whereas treatment with 10 μg/ml STW3-VI decreased the TNF-α release induced by the higher concentration of H_2_O_2_ (4 mM) by 20% (Figure 2C). The pre-treatment of mouse SIM-A9 microglia with desipramine did not reduce the H_2_O_2_-induced TNF-α release (Figures 2B, C).

### Protective Effects of STW3-VI Against H_2_O_2_-Induced ROS Production in SIM-A9 Microglia

**FIGURE 3:**
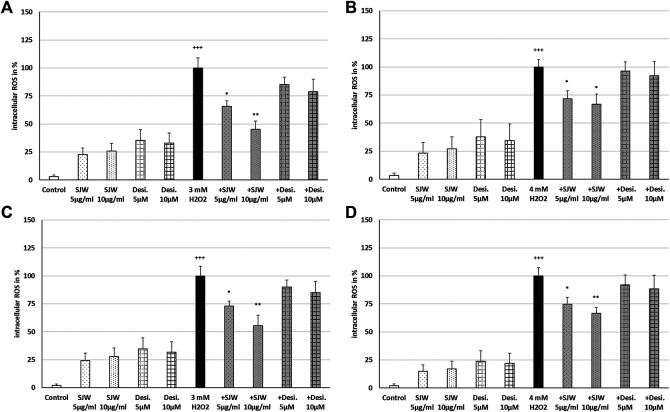
Effects of 24 h **(A,B)** or 48 h **(C,D)** pre-treatment with STW3-VI [St. John’s wort (SJW)] or desipramine (desi.) and afterwards 24 h co-incubation with 3 **(A,C)** or 4 mM H_2_O_2_ **(B,D)** on the intracellular ROS level in mouse SIM-A9 microglia. Data are given as mean + SEM; significance (by T-TEST): *p ≤ 0.05, **p ≤ 0.01, ***p ≤ 0.001 vs. 3 or 4 mM H_2_O_2_ treated cells; ^+++^ *p* ≤ 0.001 vs. untreated control; n = 4‒6.

A key player of microglial neurotoxicity is the release of excitotoxins, including glutamate and in particular ROS (Rojo et al., 2014). When ROS production rises above the antioxidant defenses, oxidative stress occurs. In this context, we investigated the possible antioxidant/protective properties of STW3-VI or desipramine against ROS. Treatment of mouse SIM-A9 microglial cells with 3 mM or 4 mM H_2_O_2_ (100% of ROS production) significantly (*p* ≤ 0.001) increased the intracellular ROS by 96.6‒98.1% compared with the untreated control (Figures 3A–D). Incubation (24 or 48 h) of mouse SIM-A9 microglia with 5 or 10 μg/ml STW3-VI, as well as with 5 or 10 µM desipramine alone did not significantly increase intracellular ROS compared with the untreated control (Figures. 3A–D). Interestingly, pre-treatment (24 h) of mouse SIM-A9 microglia with STW3-VI (5 or 10 μg/ml) significantly decreased the H_2_O_2_-induced intracellular ROS level by 34.1% (*p* ≤ 0.05) or 54.7% (*p* ≤ 0.01) compared with 3 mM H_2_O_2_ alone (Figure 3A); on the same line, pre-treatment (24 h) of mouse SIM-A9 microglia with 5 μg/ml or 10 μg/ml STW3-VI significantly (*p* ≤ 0.05) inhibited the H_2_O_2_-induced intracellular ROS by 28.2 or 33.1% compared with 4 mM H_2_O_2_ treatment alone (Figure 3B). Moreover, pre-treatment of mouse SIM-A9 microglia with 5 or 10 μg/ml STW3-VI for a longer period (48 h), yielded similar results significantly diminishing the H_2_O_2_-induced intracellular ROS level by 26.9% (*p* ≤ 0.05) or 44.4% (*p* ≤ 0.01) in comparison with 3 mM H_2_O_2_ treatment (Figure 3C) and by 25.2% (*p* ≤ 0.05) or 33.3% (*p* ≤ 0.01) in comparison with 4 mM H_2_O_2_ treatment, however, these effects were not seen using desipramine (Figure 3D).

### STW3-VI Protects SIM-A9 Microglia Against N-Methyl-D-Aspartate or Cortisol-Induced Cytotoxicity

**FIGURE 4:**
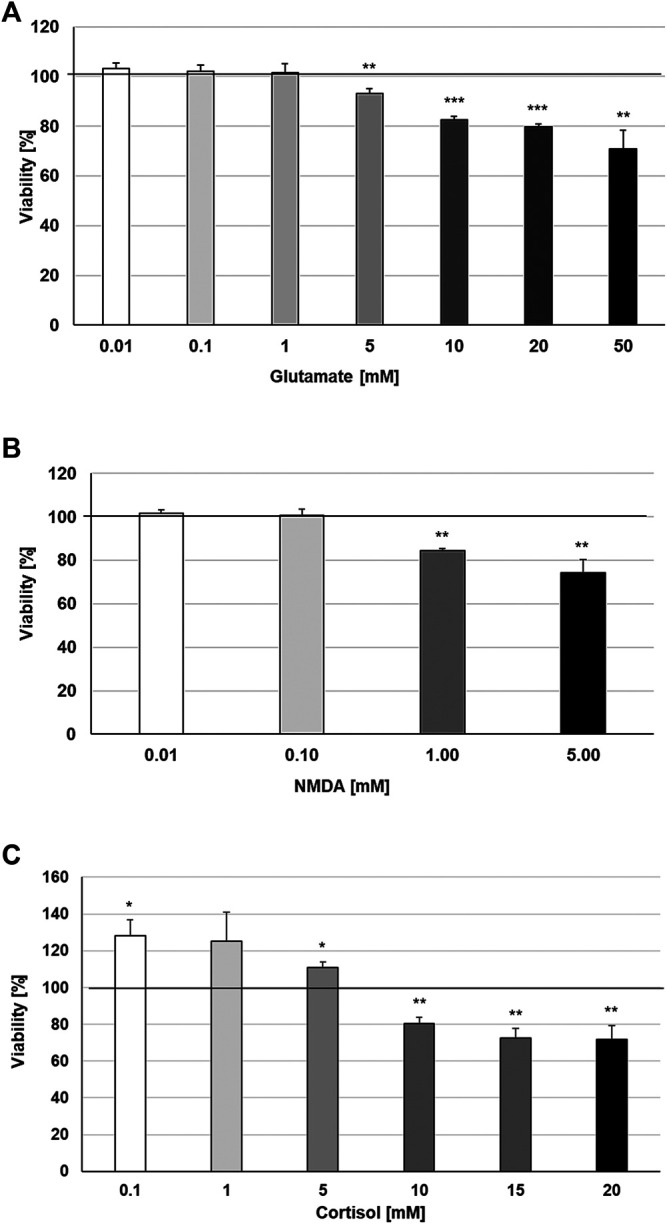
Effects of 48 h treatment with **(A)** glutamate, **(B)** N-methyl-D-aspartate or **(C)** Cortisol on the viability of mouse SIM-A9 microglia. Viability was measured by PrestoBlue^®^ assay. Values [in % viability of untreated control (control = 100% viability)] are given as mean + SEM; significance (by T-TEST): *p ≤ 0.05, **p ≤ 0.01 or ***p ≤ 0.001 vs. untreated control; n = 4.

Increases in synaptic glutamate, resulting from early inflammatory changes, are responsible for the overactivation of synaptic ionotropic receptors such as NMDA, potentially contributing to excitotoxicity (Haroon et al., 2017). The effects of TNF-α on NMDA receptors are not yet well defined, although NMDA receptor blockers are known to confer neuroprotection against glutamate toxicity (Zou and Crews, 2005). In this context, we investigated the possible protective properties of STW3-VI against glutamate-, NMDA- or cortisol-induced cytotoxicity in mouse SIM-A9 microglia. We investigated the cytotoxic effects of various concentrations of glutamate, NMDA, and cortisol. We found that concentrations of 0.01‒1 mM glutamate were not cytotoxic for mouse SIM-A9 microglia, whereas 5, 10, 20, or 50 mM glutamate significantly (*p* ≤ 0.01−0.001) reduced the viability by 6.8, 17.5, 20.2, and 29.0% in comparison with the untreated control (Figure 4A). Treatment of mouse SIM-A9 microglia with 1.0 or 5.0 mM NMDA significantly (*p* ≤ 0.01) diminished the viability by 15.7% and 25.8% in comparison with the untreated control, whereas 0.01 or 0.1 mM NMDA were not cytotoxic (Figure 4B). After treatment of mouse SIM-A9 microglia with 10.0, 15.0 or 20.0 mM cortisol, the viability was significantly decreased by 19.4 (*p* ≤ 0.01), 27.3 (*p* ≤ 0.01) and 28.4% (*p* ≤ 0.01), while lower concentrations of 0.1 or 5.0 mM increased significantly the viability by 28.4 and 11.0% when compared with the untreated control (Figure 4C).

**FIGURE 5:**
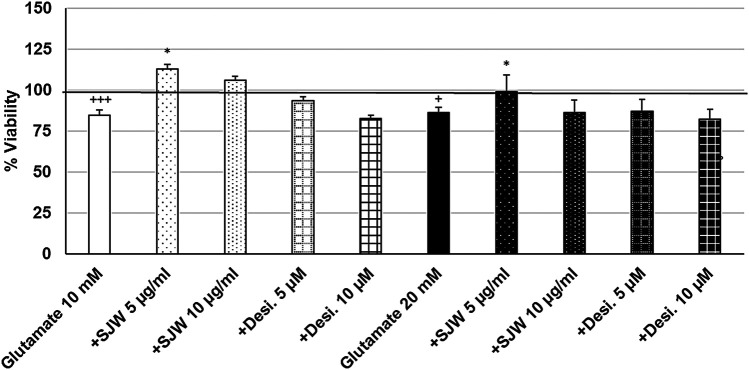
Effects of 48 pre-treatment with STW3-VI [St. John’s wort (SJW)] or desipramine and afterwards 24 h co-incubation with glutamate (10 or 20 mM) on viability of mouse SIM-A9 microglia. Viability was measured by PrestoBlue^®^ assay. Values [in % viability of untreated control (control = 100% viability)] are given as mean + SEM; significance (by T-TEST): *p < 0.05, vs. glutamate; ^+^ *p* < 0.05, +++*p* < 0.001 vs. untreated control (100%); n = 4‒12.

To investigate the possible protective properties of STW3-VI against glutamate-, NMDA- or cortisol-induced cytotoxicity in mouse SIM-A9 microglia, we determined the viability after pre-treatment with STW3-VI (5 or 10 μg/ml) or desipramine (5 or 10 µM) followed by co-incubation (24 h) with glutamate (10 or 20 mM), NMDA (1 or 5 mM) or cortisol (10 or 15 mM). Our data show that the viability of mouse SIM-A9 microglia was significantly (*p* ≤ 0.001) reduced by 15.3 and 13.9% after treatment with 10 or 20 mM glutamate, when compared to untreated control (Figure 5). Interestingly, pre-treatment (48 h) of mouse SIM-A9 microglia with 5 μg/ml STW3-VI—in contrast to desipramine - inhibited the glutamate-induced cytotoxicity by 28.3% (10 mM, *p* ≤ 0.05) or 13.2% (20 mM, *p* ≤ 0.05) in comparison with 10 or 20 mM glutamate alone (Figure 5).

**FIGURE 6:**
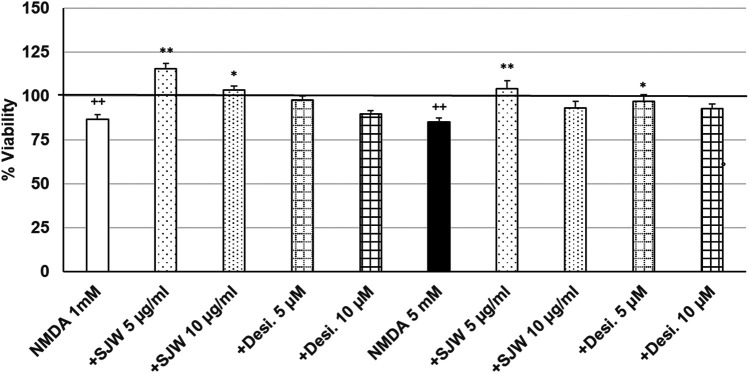
Effects of 48 pre-treatment with STW3-VI [St. John’s wort (SJW)] or desipramine and afterwards 24 h co-incubation with N-methyl-D-aspartate (NMDA) (1 or 5 mM) on viability of mouse SIM-A9 microglia. Viability was measured by PrestoBlue^®^ assay. Values [in % viability of untreated control (control = 100% viability)] are given as mean + SEM; significance (by T-TEST): *p < 0.05, **p < 0.01, vs. NMDA ++*p* ≤ 0.01 vs. untreated control; ++*p* < 0.01 vs. untreated control (100%); n = 4‒12.

After 24 h treatment (24 h) of mouse SIM-A9 microglia with NMDA (1 or 5 mM), the viability was significantly (*p* ≤ 0.01) decreased by 13.3 or 15.0% compared with untreated control (Figure 6). Pre-treatment (48 h) of mouse SIM-A9 microglia with 5 or 10 μg/ml STW3-VI—in contrast to desipramine - significantly arrested the NMDA-induced cytotoxicity by 28.8% (*p* ≤ 0.01) or 16.5% (*p* ≤ 0.05) compared with 1 mM NMDA treatment (Figure 6). The cytotoxic effect of 5 mM NMDA was significantly (*p* ≤ 0.01) inhibited by 18.9%, when mouse SIM-A9 microglia were pre-treated (48 h) with 5 μg/ml STW3-VI or by 12% (*p* ≤ 0.05) with desipramine 5 µM (Figure 6).

**FIGURE 7:**
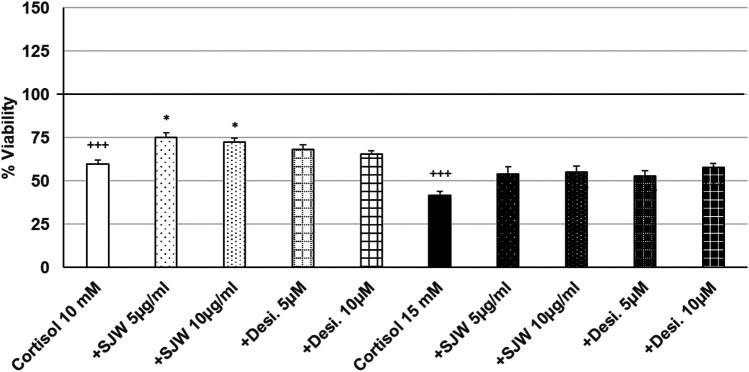
Effects of 48 pre-treatment with STW3-VI [St. John’s wort (SJW)] or desipramine and afterwards 24 h co-incubation with cortisol (10 or 15 mM) on viability of mouse SIM-A9 microglia. Viability was measured by PrestoBlue^®^ assay. Values [in % viability of untreated control (control = 100% viability)] are given as mean + SEM; significance (by T-TEST): *p ≤ 0.05, vs. cortisol; +++*p* ≤ 0.001 vs. untreated control (100%); n = 4‒12.

Treatment of mouse SIM-A9 microglia with 10 mM or 15 mM cortisol significantly (*p* ≤ 0.001) reduced the viability by 40.5% and 58.4% compared with the untreated control (Figure 7). Pre-treatment (48 h) of mouse SIM-A9 microglia with 5 or 10 μg/ml of STW3-VI—in contrast to desipramine - decreased the cortisol-induced cytotoxicity by 15.5% (*p* ≤ 0.05) and 12.9% (*p* ≤ 0.05) compared with cortisol (10 mM) treatment (Figure 7).

### Stimulatory Effects of STW3-VI on the Migratory Capacity of SIM-A9 Microglia

**FIGURE 8:**
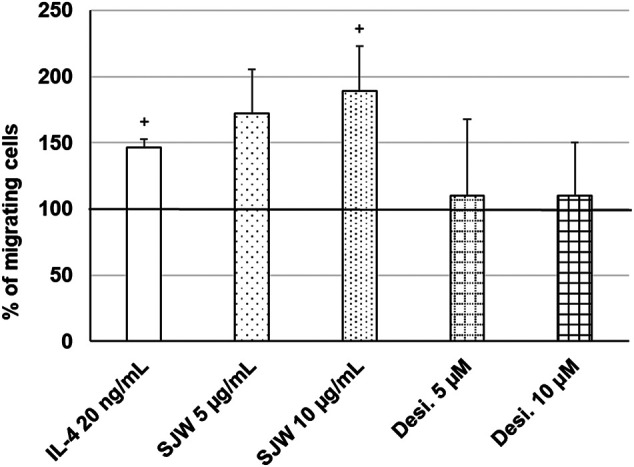
Effects of 24 h treatment with STW3-VI [St. John’s wort (SJW)], desipramine (desi.), or IL-4 as positive control on migratory capacity of mouse SIM-A9 microglia. Quantification of migrated cells/cm^2^ in % of negative control (100%) expressed as mean + SEM; significance (by T-TEST): ^+^ *p* ≤ 0.05 vs. untreated control; n = 3‒5 independent experiments.

Important functions of microglia in the CNS include synaptic pruning, phagocytosis of neurons, their debris and migration to the sites of injury (Xavier et al., 2014; Zhang et al., 2016). Thus, we investigated the effect of STW3-VI (5 or 10 μg/ml) or desipramine (5 or 10 µM) on the migratory capacity of mouse SIM-A9 microglia using the transwell migration assay. The results indicate that the treatment (24 h) of mouse SIM-A9 microglia with STW3-VI (10 μg/ml) significantly (*p* ≤ 0.05) stimulated the migratory capacity by 2.0-fold compared with untreated control (Figure 8). In contrast, the treatment of mouse SIM-A9 microglia with desipramine did not stimulate the migratory capacity (Figure 8). IL-4 treatment was used as a positive control of migration (Figure 8).

### Stimulatory Effects of STW3-VI on the Phagocytic Capacity of SIM-A9 Microglia

**FIGURE 9:**
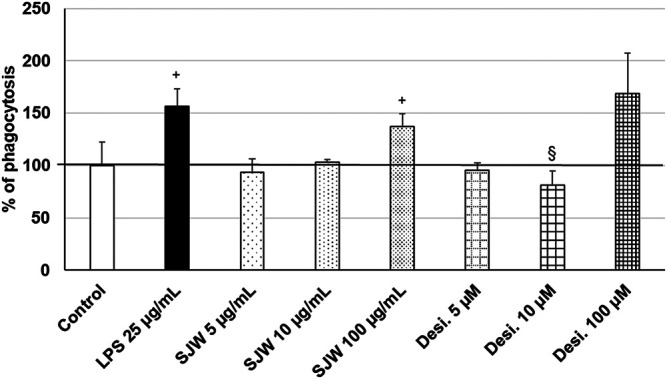
Effects of 48 h treatment with STW3-VI [St. John’s wort (SJW], desipramine (desi.), or lipopolysaccharide as positive control and afterwards for 24 h with FBS-coated FITC-fluorescent microspheres, on phagocytic capacity of mouse SIM-A9 microglia. Quantification of phagocytosis/DAPI in % of untreated control (100%) expressed as mean + SEM; significance (by T-TEST): ^+^ *p* ≤ 0.05 vs. untreated control; ^§^ *p* ≤ 0.05 vs. SJW 100 µg/ml; n = 3‒6.

Treatment (48 h) of mouse SIM-A9 microglia with a very high dose of 100 μg/ml STW3-VI—in contrast to desipramine - significantly (*p* ≤ 0.05) increased the phagocytic capacity by 40% compared with the untreated control (Figure 9). Additionally, a high dose of 100 µM desipramine only insignificantly increased the phagocytic capacity. Treatment (48 h) of mouse SIM-A9 microglia with a well-known pro-phagocytic stimulus LPS significantly (*p* ≤ 0.05) increased the phagocytosis by 55% (Figure 9).

## Discussion

*Hypericum perforatum* L. [St. John’s wort, (SJW)] is widely used as an herb for the therapy of various diseases since ancient times. STW3-VI contains a dry extract of SJW that is commercially available as Laif^®^900. It is particularly used for the treatment of depression (Butterweck et al., 1998; Butterweck, 2003; Linde et al., 2008; Grundmann et al., 2010). In this context, the whole extract must be considered with antidepressant properties, because a synergistic effect of hypericin, flavonoids, and hyperforin is responsible for its antidepressant efficacy (Butterweck and Schmidt, 2007). Two of the causes which trigger neurodegenerative diseases and major depressive disorder (MDD) are oxidative stress and inflammation (Fischer and Maier, 2015; Saitgareeva et al., 2020). Recently, we have reported that STW3-VI protects differentiated HT-22 hippocampal neurons against the glutamate- or NMDA-induced cytotoxicity (Bonaterra et al., 2018). Furthermore, STW3-VI has shown anti-inflammatory effects and stimulated outgrowth of neurites, which is a characteristic of neuronal plasticity and neurogenesis (Bonaterra et al., 2018). Additionally, we have provided evidence that STW3-VI is an agent with anti-inflammatory properties on LPS-stimulated human macrophages as an *in vitro* model of resident macrophages (Bonaterra et al., 2018). Microglia are long-living and self-renewing cells and are considered to be resident macrophages in the CNS (Yirmiya et al., 2015; Laskaris et al., 2016; Szepesi et al., 2018). Microglia are essential for tissue repair processes and the maintenance of homeostasis during infectious diseases, ischemic lesions, and tumor growth, which can activate the over-expression of proinflammatory cytokines, e.g., TNF-α, IL-1β as well as neurotoxic radicals, e.g., ROS, nitric oxide (Singhal and Baune, 2017). Chronic stress induces the release of inflammatory factors such as TNF-α by microglia, which causes inflammatory reactions (Zhang et al., 2018). The chronic over-activation of microglia and oxidative stress are suggested to induce neurodegenerative diseases and major depression. However, ROS produced by either microglia or the surrounding environment not only affect neurons but also modulate microglial activity (Rojo et al., 2014; Wenzel et al., 2020). These proinflammatory cytokines and ROS may induce neuroinflammatory and neurodegenerative processes, and the following illnesses such as e.g., Parkinson’s, Alzheimer’s diseases, schizophrenia, bipolar disorders, and MDD (Patel, 2013; Kurt et al., 2020). The increase of ROS levels during the state of oxidative stress in the brain is suggested as a key factor to induce activation and phenotypic changes in microglia and astrocytes (Pawate et al., 2004; Rivas-Arancibia et al., 2010). Excessive generation of ROS causes neuronal damage via apoptosis, and consequently, the release of cytosolic factors that activate neighboring astrocytes and microglia (Chen et al., 2020). Astrocytes and microglia respond by the release of proinflammatory cytokines, e.g., TNF-α, as well as a high amount of ROS, thus further promoting the inflammatory response and exacerbating the neuronal damage (Fischer and Maier, 2015), leading to an initiation of a vicious circle, where TNF-α can increase the generation and release of a huge amount of ROS and trigger persistent activation of glial cells and neurodegeneration (Fischer and Maier, 2015). We found that oxidative stress simulated by the treatment of mouse SIM-A9 microglia with H_2_O_2_ induces an increased release of TNF-α. However, pre-treatment with STW3-VI protected mouse SIM-A9 microglia from the pro-inflammatory effect of H_2_O_2_ and inhibited the release of TNF-α. Various studies suggest that cytokines as IL-1β and TNF-α lead to microglial activation that generates harmful effects on cell survival and decrease of neurite growth, consequently reducing hippocampal neurogenesis (Monje et al., 2003; Farrell et al., 2016; Wang et al., 2018). These anti-inflammatory effects of STW3-VI on mouse SIM-A9 microglia after pro-inflammatory induction by oxidative stress are shown here for the first time. Protection against oxidative stress and detoxification of ROS is fundamental for all cells to survive. Living organisms have developed a variety of defense mechanisms to provide a balance between the generation and elimination of ROS. In this context, we found that STW3-VI has protective properties against the harmful effects of increased intracellular ROS, that was induced by the treatment of mouse SIM-A9 microglia with H_2_O_2_. Among other mechanisms, the anti-depressant action of STW3-VI seems to be mediated by the link between the immune-/neuroendocrine system and oxidative defense and may protect against the increase of intracellular ROS or TNF-α release i.e., oxidative stress and inflammation. Thus, we demonstrate for the first time, that STW3-VI can inhibit the detrimental effect of TNF-α and oxidative stress to microglia as well as possibly neighboring cells and, thus, consequently protective effects of STW3-VI against neuroinflammatory processes. Pro-inflammatory cytokines can activate the HPA axis leading to hypercortisolism and increased glucocorticoid receptor resistance, both mechanisms are involved in the etiology of MDD (Cohen et al., 2012). Additionally, pro-inflammatory cytokines modulate the kynurenine pathway of tryptophan and enhance the synthesis of the neurotoxic NMDA receptor agonist quinolinic acid and 3-hydroxykynurenine with detrimental effects on brain function (Dantzer et al., 2011). Alterations of the glutamatergic system, especially the receptors of glutamate and NMDA, are involved in the pathology of neurodegenerative diseases, as well as MDD (Serafini et al., 2013; Dantzer and Walker, 2014; Haroon et al., 2016). In this regard, inflammation and glutamate toxicity play a major role in a variety of neurological disorders like anxiety, depression, etc. (Tokarski et al., 2008; Haroon et al., 2016). Glutamate, an excitatory and cytotoxic neurotransmitter in the CNS, can accumulate in the brain and, thus, can initiate and/or aggravate neurodegenerative diseases (Sun et al., 2010; Hu et al., 2012). STW3-VI has been previously shown to be protective against glutamate/NMDA-induced cytotoxicity in (un)differentiated hippocampal HT-22 cells (Breyer et al., 2007; Bonaterra et al., 2018). In this context we now show, that the pre-treatment of mouse SIM-A9 microglia with STW3-VI protects against cytotoxic concentrations of glutamate or NMDA. Microglia have numerous receptors, that can bind neurotransmitters related to stress, such as glutamate (Hellwig et al., 2016). Excessive glutamate release and hence glutamate receptor activation can induce neuronal and microglial cell death (Szepesi et al., 2018). Chronic stress can cause the activation of microglia and neurons, and the pharmacological inhibition of NMDA receptors may thus inhibit the activation of both cell types (Wendt et al., 2016).

Additionally, serotonergic dysfunctions and cortisol dysregulation may be responsible for the symptoms of depression (Maes et al., 2009). Moreover, depression symptoms have been associated with increases of salivary cortisol levels as well as hypercortisolemia and with chronic activation of the HPA axis, which may be increased by psychosocial stressors (Lopez-Duran et al., 2015). Therefore, corticosterone is utilized as a marker for assessing stress in studies of humans and animals alike (Costantini et al., 2011; Kalliokoski et al., 2019); additionally, elevated levels of pro-inflammatory cytokines such as TNF-α, IL-1β, and IL-6 may serve as biological markers of anxiety disorders (Sevastre-Berghian et al., 2018). Further possible mechanisms include the interactions of cortisol with inflammatory cytokines, neurotransmitters, which together may exert detrimental effects on the capacity of cognition and memory (Ouanes and Popp, 2019), representing characteristic symptoms of depression. Pro-inflammatory cytokines, as well as high levels of cortisol may promote oxidative stress and also neurotoxic effects on the hippocampus (Sudheimer et al., 2014; Ouanes and Popp, 2019) and are linked e.g., to hippocampal atrophy (Tatomir et al., 2014), which may be recovered after normalization of the cortisol levels (Starkman et al., 1999). In this context, we found that STW3-VI counteracts the cytotoxic effect of cortisol on mouse SIM-A9 microglia. This effect of STW3-VI is suggested as protective against cortisol-induced stress, followed by neurotoxicity in CNS, and consequently may be beneficial in the treatment of depressive disorders.

In the CNS, the renewal of new neuronal populations is limited to certain brain regions such as the subgranular zone of the hippocampus (Kaplan and Hinds, 1977); thus, the use of natural products that promote neurogenesis and plasticity may be applicable to treat depressive stages. In this regard, treatment of hippocampal neurons with STW3-VI enhanced the growth of neurites, suggesting that these neurotrophic properties might play a role in its positive effects on depression (Bonaterra et al., 2018). Our results concerning the protective effects of STW3-VI against glutamate- and NMDA-induced toxicity on microglia and neurons may be a validation of the positive and protective properties of STW3-VI in neurodegenerative diseases and MDD as well. Other, important functions of microglia in the CNS include migration, synaptic pruning, and phagocytosis of dead neuronal cells and other cell debris (Xavier et al., 2014; Zhang et al., 2016). Microglia react to injury with phenotypic changes, proliferation, migration, and release of inflammatory and anti-inflammatory cytokines (Ramirez et al., 2017). As well, nucleotides released by damaged neurons can trigger inflammatory responses in microglia (Ramirez et al., 2017) and consequently, can up-regulate its purinergic receptors, activating their phagocytic and migratory activity (Koizumi et al., 2007; Ohsawa et al., 2007; Wu et al., 2007). Phagocytosis is a critical function in early neural development, homeostasis, and repair mechanisms (Galloway et al., 2019). As such, modulating phagocytic processes may be a way to develop novel therapeutics that promote repair and regeneration in the CNS (Galloway et al., 2019). Accumulation of activated microglia (microgliosis) around damaged neurons is a common pathological characteristic of various neurological disorders (Takeuchi 2010). Potential targets against neurodegenerative diseases caused by activated microglia are the therapeutic blockade of glutamate receptors and the inhibition of the inflammatory and oxidative activation of microglia (Takeuchi 2010). However, some clinical trials failed, because of side effects, e.g., Edaravone - a clinically approved ROS inhibitor, used to help people recover from stroke - induced severe renal failure (Takeuchi 2010). Nevertheless, the use of low toxicity therapeutics without or with low side effects have hopeful perspectives. In this context, by using a transmigration assay, we were able to show for the first time, that STW3-VI—in contrast to desipramine - activated the migration of mouse SIM-A9 microglia. Additionally, we found that STW3-VI—in contrast to desipramine - activated the phagocytic capacity in spontaneously differentiated mouse SIM-A9 microglia. This is not consistent with earlier data showing that hyperforin treatment reduced zymosan phagocytosis in BV2 and N11 microglia cells (Kraus et al., 2010), which are virally immortalized cells (Righi et al., 1989; Blasi et al., 1990; Timmerman et al., 2018). Furthermore, extracts often show effects different from their isolated constituents. Interestingly, we found an increased phagocytic activity by using obviously cytotoxic concentrations of STW3-VI, which may be explained by the fact that the primary functional effector state of microglia is primed for phagocytosis, but not cytotoxicity (Adams et al., 2015). Thus, under cytotoxic conditions, microglia increase their phagocytic capacity (Adams et al., 2015). We here confirm this assumption by the measurement of intracellular phagocytized particles normalized to the cell quantity, and we were able to show that incubation with STW3-VI increased the number of phagocyted microparticles/cell.

Chemotaxis of phagocytes to the inflammatory site is the first step that is decisive for the activation of the host defense (Hsu et al., 2003). Thus, these properties may be associated with a positive effect of STW3-VI to induce migration of microglia to injured sites with improved phagocytosis of damaged cells such as neurons.

## Conclusion

Our data furnish proof that STW3-VI, in contrast to desipramine, has anti-inflammatory and anti-oxidative properties and protects SIM-A9 microglia cells against NMDA- or glutamate-induced cytotoxicity, in addition to improvement of their migratory and phagocytic capacity. These protective features may be also beneficial in the STW3-VI treatment of patients with depressive disorders. However, further investigations are required to understand the molecular mechanism of action and target(s) of STW3-VI *in vitro* and *in vivo*.

## Data Availability

The original contributions presented in the study are included in the article/Supplementary Material, further inquiries can be directed to the corresponding author.
