# Supplementary material for: In Vitro Effects of St. John’s Wort Extract Against Inflammatory and Oxidative Stress and in the Phagocytic and Migratory Activity of Mouse SIM-A9 Microglia
Source: Front Pharmacol. 2020 Dec 3;11:603575. doi: 10.3389/fphar.2020.603575 (PMC7898673; doi:10.3389/fphar.2020.603575)
Supplement: Supplementary file 1 [file datasheet1.pdf]

==== Shimadzu LCsolution Analysis Report ===

Steigerwald Arzneimittelwerk GmbH - Qualitätskontrolle

C:\LabSolutions\Data\Littmann QK0106\Hypericin_PhEur_L40807_19.lcd

Shimadzu solution Version: 5.42 SP2
Instrument Name: QK0106
Acquired by: F. Littmann
Data Acquired: 07.08.2014 20:57:59
Injection Volume: 20 uL
Original Method File Name: C:\LabSolutions\Data\Littmann QK0106\Gehalt_Hypericin_PhEUR_QK0106.lcm
Original Batch File Name: C:\LabSolutions\Data\Littmann QK0106\Hypericin_PhEur_L40807.lcb

Sample Name: Johanniskraut-Trockenextrakt (3-6:1) #14-0155 [71.59 mg]
Sample ID: Mischprobe 3
Description:

<Chromatogram>

C:\LabSolutions\Data\Littmann QK0106\Hypericin_PhEur_L40807_19.lcd

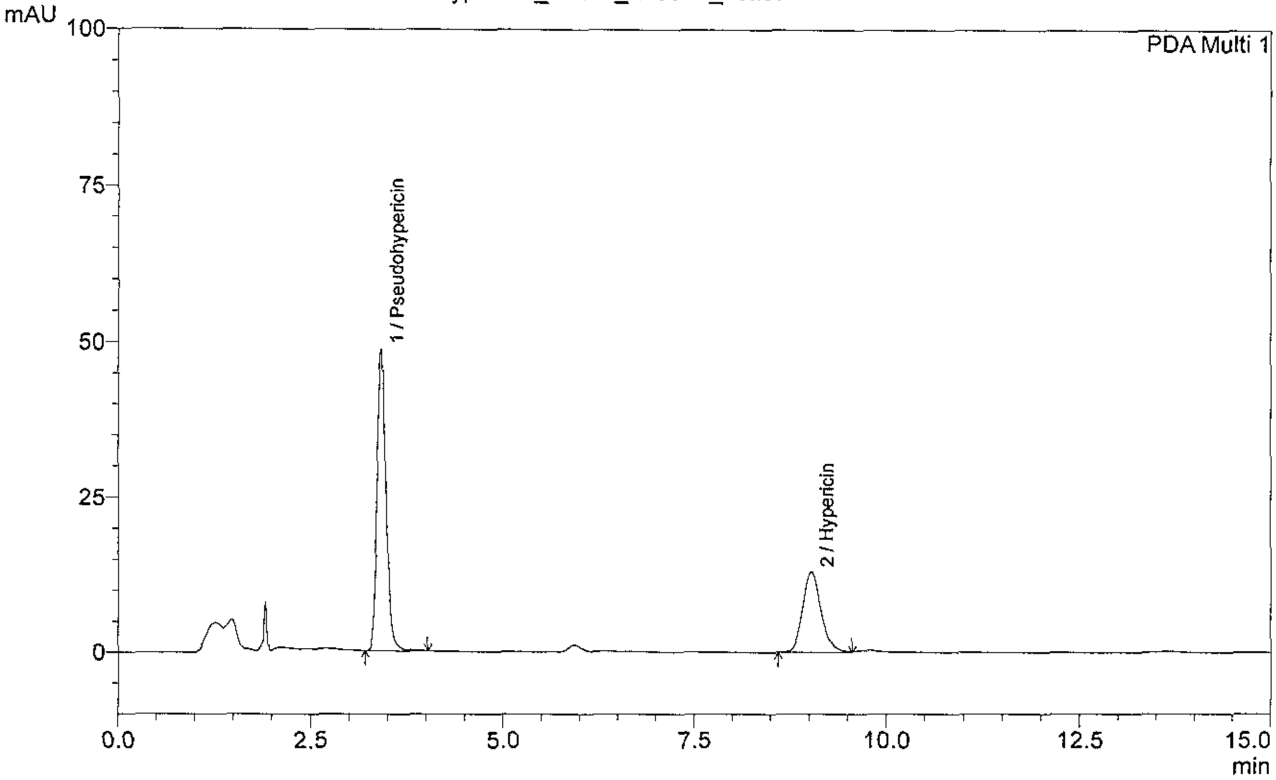

1 590nm,4nm / 07.08.2014

<Results>

PeakTable C:\LabSolutions\Data\Littmann QK0106\Hypericin_PhEur_L40807_19.lcd
PDA Ch1 590nm

Peak#	Name	Ret. Time	Area	Mark
1	Pseudohypericin	3.407	397117	
2	Hypericin	9.023	219187	H
Total			616304	
