# Supplementary material for: In Vitro Effects of St. John’s Wort Extract Against Inflammatory and Oxidative Stress and in the Phagocytic and Migratory Activity of Mouse SIM-A9 Microglia
Source: Front Pharmacol. 2020 Dec 3;11:603575. doi: 10.3389/fphar.2020.603575 (PMC7898673; doi:10.3389/fphar.2020.603575)
Supplement: Supplementary file 2 [file datasheet2.pdf]

==== Shimadzu LCsolution Analysis Report ===

Steigerwald Arzneimittelwerk GmbH - Qualitätskontrolle

C:\LabSolutions\Data\Littmann QK0106\Hyperforin_Flavonoide_L40812_19.lcd

Shimadzu solution Version	: 5.42 SP2
Instrument Name	: QK0106
Acquired by	:F. Littmann
Data Acquired	:13.08.2014 02:51:58
Injection Volume	:10 uL
Original Method File Name	: C:\LabSolutions\Data\Littmann QK0106\Gehalt_Hyperforin_Flavonoide_PhEUR_(
Original Batch File Name	: C:\LabSolutions\Data\Littmann QK0106\Hyperforin_Flavonoide_L40812.lcb

Sample Name	: Johanniskraut-Trockenextrakt (3-6:1) #14-0155 [187.98 mg]
Sample ID	: Mischprobe 1
Description	:

<Chromatogram>

Chromatogram
Johanniskraut-Trockenextrakt (3-6:1) #14-0155 [187.98 mg] C:\...\Data\Littmann QK0106\Hyperforin_Flavonoide_L40812_19.lcd

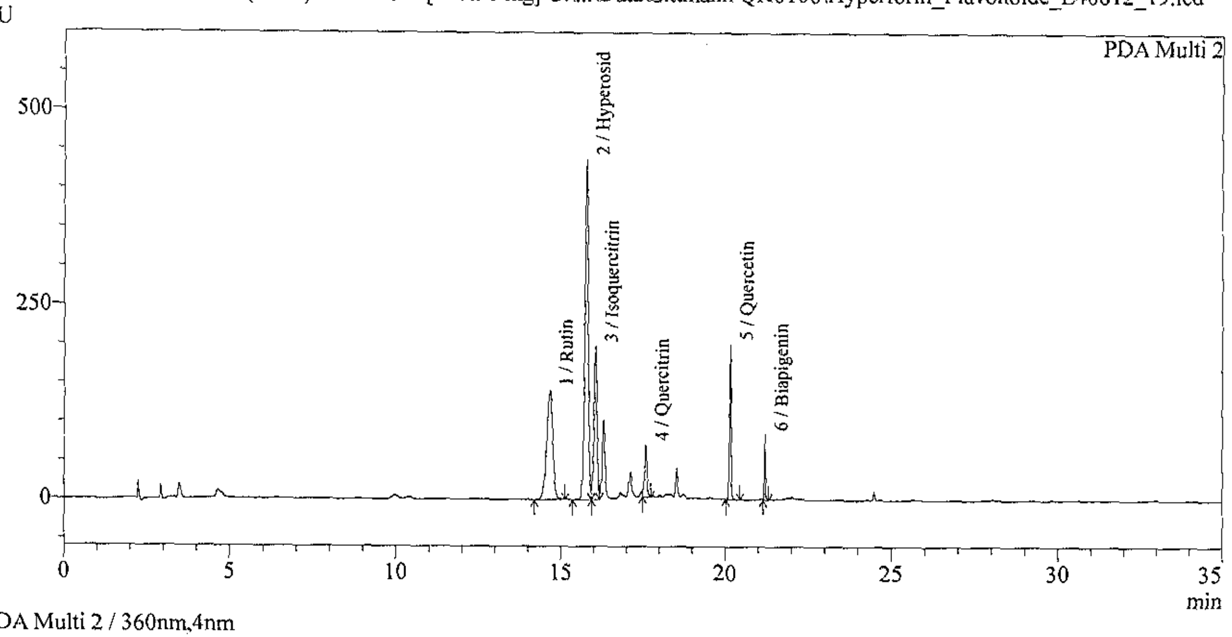

1 PDA Multi 2 / 360nm,4nm

<Results>

PeakTable C:\LabSolutions\Data\Littmann QK0106\Hyperforin_Flavonoide_L40812_19.lcd
PDA Ch2 360nm

Peak#	Name	Ret. Time	Area	Group Name	Mark
1	Rutin	14.669	1823806	Flavonoide	H
2	Hyperosid	15.775	3184394	Flavonoide	H
3	Isoquercitrin	16.055	1272768	Flavonoide	H
4	Quercitrin	17.592	353481	Flavonoide	V
5	Quercetin	20.148	699552	Flavonoide	
6	Biapigenin	21.199	212396	Flavonoide	V
Total			7546395		

Grouping Results C:\LabSolutions\Data\Littmann QK0106\Hyperforin_Flavonoide_L40812_19.lcd
PDA

Group#	Group Name	Area
1	Flavonoide	7546395
2	Hyperforine	647383
Total		8193779

==== Shimadzu LCsolution Analysis Report ===

Steigerwald Arzneimittelwerk GmbH - Qualitätskontrolle

C:\LabSolutions\Data\Littmann QK0106\Hyperforin_Flavonoide_L40812_19.lcd

Shimadzu solution Version	: 5.42 SP2
Instrument Name	: QK0106
Acquired by	:F. Littmann
Data Acquired	:13.08.2014 02:51:58
Injection Volume	:10 uL
Original Method File Name	: C:\LabSolutions\Data\Littmann QK0106\Gehalt_Hyperforin_Flavonoide_PhEUR_QK
Original Batch File Name	: C:\LabSolutions\Data\Littmann QK0106\Hyperforin_Flavonoide_L40812.lcb

Sample Name	: Johanniskraut-Trockenextrakt (3-6:1) #14-0155 [187.98 mg]
Sample ID	: Mischprobe 1
Description	:

<Chromatogram>

Chromatogram
Johanniskraut-Trockenextrakt (3-6:1) #14-0155 [187.98 mg] C:\...\Data\Littmann QK0106\Hyperforin_Flavonoide_L40812_19.lcd

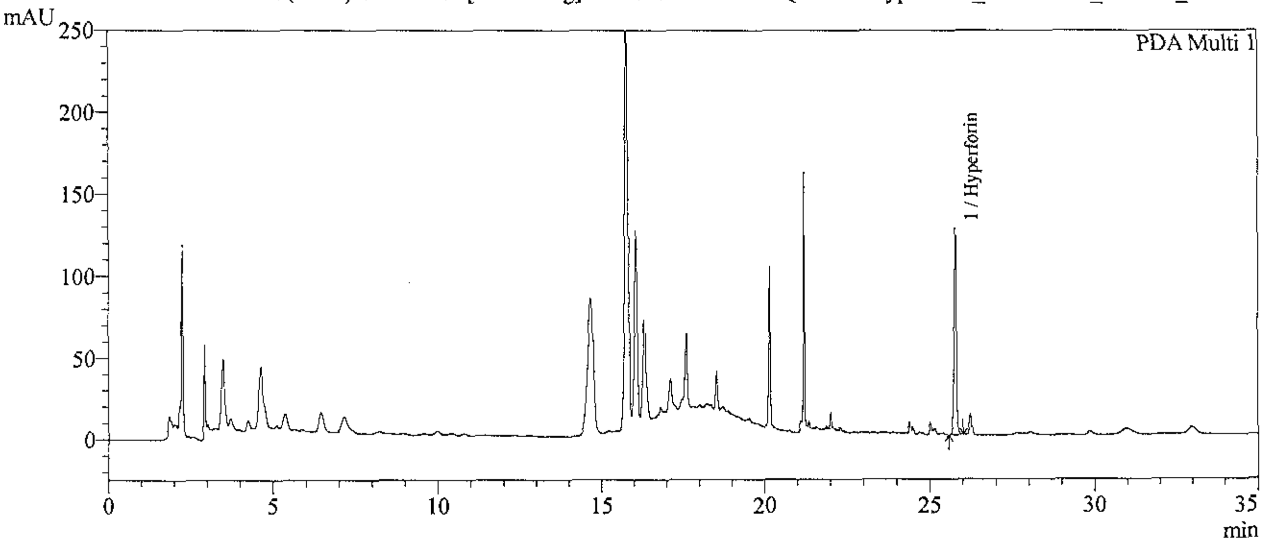

1 PDA Multi 1 / 275nm,4nm

<Results>

PeakTable C:\LabSolutions\Data\Littmann QK0106\Hyperforin_Flavonoide_L40812_19.lcd
PDA Ch1 275nm

Peak#	Name	Ret. Time	Area	Mark	Group Name
1	Hyperforin	25.782	647383		Hyperforine
Total			647383		

Grouping Results C:\LabSolutions\Data\Littmann QK0106\Hyperforin_Flavonoide_L40812_19.lcd
PDA

Group#	Group Name	Area
1	Flavonoide	7546395
2	Hyperforine	647383
Total		8193779
