# Supplementary figures and images for: In Vitro Effects of St. John’s Wort Extract Against Inflammatory and Oxidative Stress and in the Phagocytic and Migratory Activity of Mouse SIM-A9 Microglia
Source: Front Pharmacol. 2020 Dec 3;11:603575. doi: 10.3389/fphar.2020.603575 (PMC7898673; doi:10.3389/fphar.2020.603575)

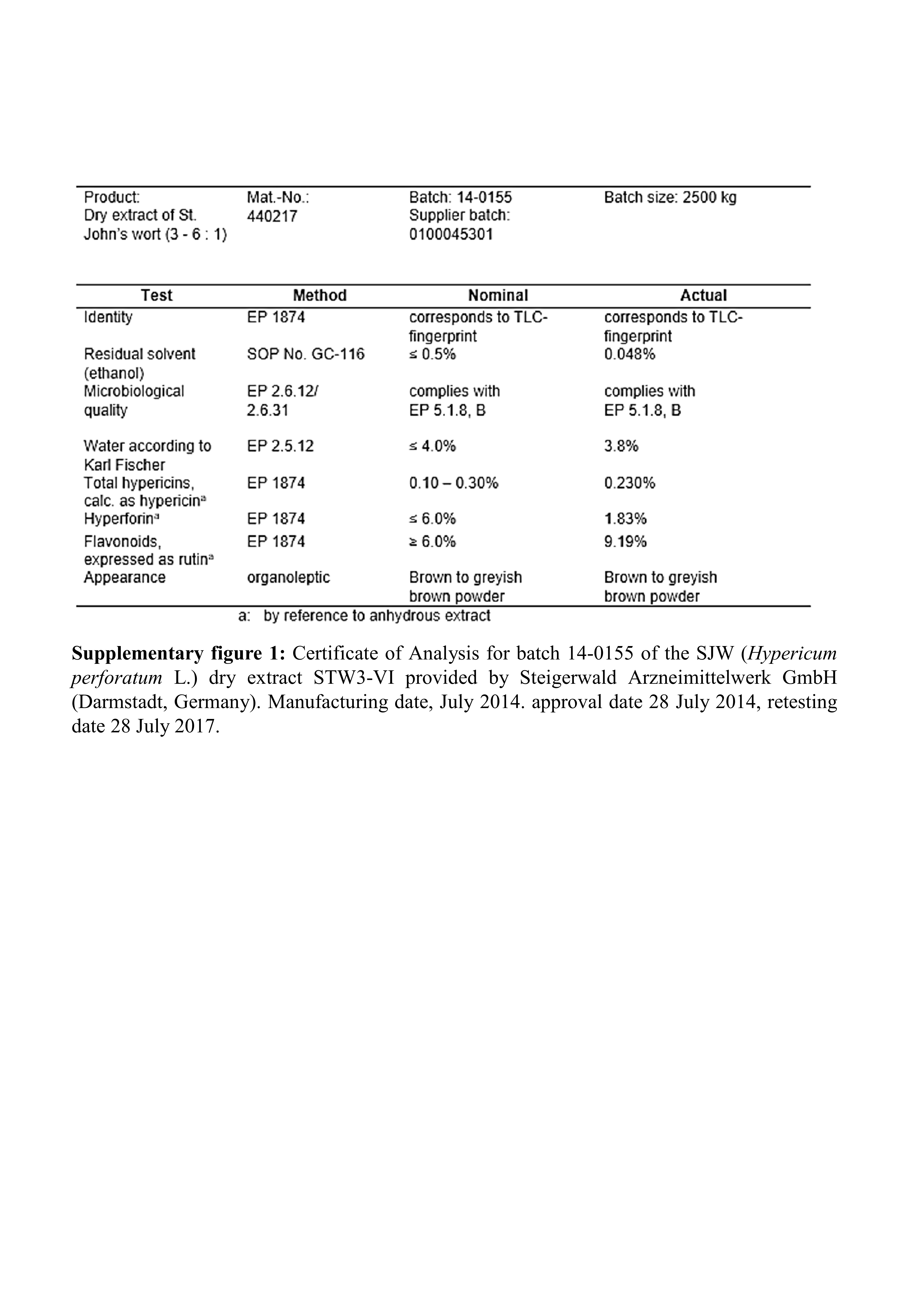

Supplement: Supplementary file 3 [file image1.tif]
